# RFWD3 Participates in the Occurrence and Development of Colorectal Cancer *via* E2F1 Transcriptional Regulation of BIRC5

**DOI:** 10.3389/fcell.2021.675356

**Published:** 2021-10-12

**Authors:** Fenghua Xu, Zhifeng Xiao, Liqin Fan, Guangcong Ruan, Yi Cheng, Yuting Tian, Minjia Chen, Dongfeng Chen, Yanling Wei

**Affiliations:** Department of Gastroenterology, Daping Hospital, Army Medical University (Third Military Medical University), Chongqing, China

**Keywords:** colorectal cancer, RFWD3, BIRC5, cell proliferation, cell apoptosis

## Abstract

**Objectives:** Colorectal cancer (CRC) is one of the most common human malignancies. It was reported that the alterations in the DNA damage response (DDR) pathways are emerging as novel targets for treatment across different cancer types including CRC. RFWD3 plays a critical role in replication protein A (RPA)-mediated DNA damage in cancer cells. More importantly, RFWD3 can response to DNA damage by positively regulating p53 stability when the G1 cell cycle checkpoint is activated. However, the functional significance of RFWD3 in CRC has not been reported in the existing documents.

**Materials and Methods:** Here, we revealed high expression of RFWD3 in CRC tissues by IHC analysis and The Cancer Genome Atlas (TCGA) database. Besides, overexpression of RFWD3 in CRC cell lines was also confirmed by qRT-PCR and western blot assay. The Celigo cell counting method and wound-healing/transwell migration assay were applied to evaluate CRC cell proliferation and migration. The tumor growth indicators were quantified in nude mice xenografted with shRFWD3 and shCtrl RKO cells.

**Results:** The results indicated that RFWD3 knockdown restricted CRC development *in vitro* and *in vivo*. In exploring the downstream mechanism of RFWD3’s action, we found that RFWD3 could transcriptionally activate BIRC5 by interacting with E2F transcription factor 1 (E2F1). Accordingly, we identified BIRC5 as a downstream gene of RFWD3 regulating CRC. Subsequent loss- and gain- of function experiments demonstrated that upon overexpressing BIRC5 in RKO cells with down-regulated RFWD3, the inhibitory effects of cell proliferation, migration and colony formation could be reversed, while the capacity of cell apoptosis was ameliorated, suggesting that the effects of RFWD3 depletion was mainly due to BIRC5 suppression.

**Conclusion:** Taken together, this study revealed that RFWD3 participates in the occurrence and development of colorectal cancer *via* E2F1 transcriptional regulation of BIRC5.

## Introduction

Colorectal cancer (CRC), with a high level of metastasis ability, accounts for a quarter of recorded cancer deaths (Zhang et al., 2016; Xie et al., 2020). It has been reported that the 5-year survival rate of patients with metastatic CRC is less than 10% (Mcquade et al., 2017). Despite the remarkable progress made in treatment, the majority of patients are often diagnosed as advanced, resulting in terrible clinical outcomes of this malignancy (van der Stok et al., 2016). At present, the courses of CRC treatment are mainly based on surgical resection, supplemented by chemotherapy and radiotherapy as well as molecular targeted therapy (Al-Busaidi et al., 2019; Piawah and Venook, 2019; Panariello et al., 2020). As demonstrated in the context of both vascular endothelial growth factor (VEGF) and epidermal growth factor receptor (EGFR) inhibitors, gefitnib, and erlotinib were valid and valuable agents to overcome CRC (Normanno et al., 2010). Additionally, the emergence of targeted drugs including panitumab, cetuximab, and bevacizumab has made the metastasis of CRC be fundamentally controlled (Fernando, 2004). However, the improvement brought by these targeted drugs was still not satisfactory in efficacy outcomes. Therefore, novel and effective therapeutic strategy is urgently needed, which requires a better understanding of CRC pathogenesis.

Ataxia Telangiectasia-mutated (ATM) and ATM-Rad3-related (ATR) are both well-known pivotal kinases in the ubiquitin proteasome system (UPS) (Mu et al., 2007; Kitajima et al., 2018). On the other hand, RING Finger and WD Repeat Domain 3 (RFWD3) was recently identified as a phosphorylation substrate protein of the ATM/ATR (Kitajima et al., 2018). RFWD3 is found to response to DNA damage by positively regulating p53 stability when the G1 cell cycle checkpoint is activated (Fu et al., 2010). An increasing body of evidence has revealed the critical roles of RFWD3 in replication protein A (RPA)-mediated DNA damage in cancer cells (Gong and Chen, 2011; Liu et al., 2011). Furthermore, RFWD3 is responsible for replication fork restart, normal repair kinetics during replication stress, and homologous recombination (HR) at stalled replication forks (Inano et al., 2017). More intriguingly, a genome-wide association study revealed that RFWD3 was a susceptible site for malignant neoplasms, such as multiple myeloma and testicular germ cell tumor (Mitchell et al., 2016). A recent research from Zhang et al. reported that RFWD3 was more elevated in tumor samples in comparison with matched normal lung tissues and was closely linked to the clinical outcomes of patients with non-small cell lung cancer, which implied that RFWD3 exerted a promoting effect on lung carcinogenesis (Zhang et al., 2020). However, the significance and elaborate molecular mechanisms of RFWD3 in CRC have not yet been elucidated.

Here, we detected RFWD3 levels in a CRC microarray combining with analyzing RFWD3 expression data for 635 CRC and 51 normal tissues cases from TCGA, finding that CRC tissues exhibited a higher level of RFWD3 compared with normal tissues. We further examined the function of RFWD3 in CRC cell behaviors and explored possible molecular mechanism in CRC. The findings indicated that RFWD3 knockdown restricted CRC development *in vitro* and *in vivo*. Mechanically, we found that RFWD3 could transcriptionally activate BIRC5 by interacting with E2F1 and identified BIRC5 as a downstream gene of RFWD3 regulating CRC. Subsequent loss- and gain- of function assays illustrated the effects of RFWD3 depletion on CRC was mainly due to BIRC5 suppression. This study furthered our understanding to the modulatory function of RFWD3 in CRC development and identified novel potential therapeutic targets for this disease.

## Materials and Methods

### Cell Culture

All cell lines used in this study, including human normal colorectal mucosal cell line FHC, colorectal adenocarcinoma epithelial cell line DLD-1, CRC cell lines SW480, Caco2, RKO, HCT 116, and HT-29, were purchased from Cell Resource Center, Institute of Basic Medicine, Chinese Academy of Medical Sciences (Beijing, China). The cells were cultured in 1640 medium with 10% FBS, placing in a 37°C incubator containing 5% CO_2_.

### Immunohistochemistry (IHC)

A microarray including 85 CRC tissues and 61 adjacent normal tissues were provided by Shanghai Outdo Biotech Co., Ltd (Shanghai, China). All patients who provided samples signed an informed consent form and provided their relevant clinical characteristics. The tissue slides were firstly placed in the oven at 65°C for 30 min, then soaked by xylene and washed using alcohol (China National Pharmaceutical Group Co., Ltd, Beijing, China). After that, the samples were repaired with 1 × EDTA (Beyotime Biotechnology Co., Ltd, Shanghai, China) and blocked with 3% H_2_O_2_ and serum. Next, the slides were incubated with primary antibody and secondary antibody at 4°C overnight. Continuously, the slides were stained with DAB and hematoxylin (Baso DiagnosticsInc., Zhuhai, China). Finally, the slides were sealed with neutral resin (China National Pharmaceutical Group Co., Ltd, Beijing, China) and imaged under an inverted microscope (IX73, Olympus, Tokyo, Japan). Two pathologists independently and randomly examined all slides. The positive cell score was graded as 0 (0%), 1 (1–25%), 2 (26–50%), 3 (51–75%), or 4 (76–100%). The staining intensity of RFWD3 was scored as 0 (negative), 1 (weak), 2 (positive + +) and 3 (positive + + +). Immunohistochemistry (IHC) results based on the positive cell score ^∗^ the staining intensity were classified into four categories: negative (0), positive (1–4), + + positive (5–8), or + + + positive (9–12). Finally, the high and moderate expression parameters were determined by the median of IHC scores of all tissues. Antibodies used were listed in Supplementary Table 1.

### The Cancer Genome Atlas (TCGA) Database Analysis

In this study, our expression profile analysis was based on the RNAseq counts data of 635 tumor and 51 normal samples from TCGA- Colon adenocarcinoma (COAD) and TCGA- Rectum adenocarcinoma (READ). First, we searched keywords related to colon adenocarcinoma and rectum adenocarcinoma and used the GDC download tool to download RNAseq count files in batches. Subsequently, according to the barcode information of the samples, the original data files of 686 samples were split from the sample list (Supplementary materials). The original data used for subsequent statistical analysis needed to be filtered and standardized. Thus, for each gene symbol, we screened and matched the transcript counts data matrix corresponding to the mRNA. Data standardization was performed using “estimate the dispersion” method in DEseq2. Genes whose mRNA transcripts had a read count of less than 10 were pre-filtered and eliminated. We further performed a quality control by PCA (principal component analysis). For the statistical analysis of multiple pairs of samples, the log2 Fold Change (logFC) was calculated between different groups, and the filter criterion was | logFC| > log2 (1.5). Genes with a *P* value less than 0.05 calculated with Benjamini-Hochberg (BH) adjustment method were considered to be differentially expressed genes that met the null hypothesis. We eventually selected the gene with the highest FC and the smallest *P* value as the target gene of this study (Robinson and Smyth, 2007, 2008; Lund et al., 2012; McCarthy et al., 2012).

### Plasmid Construction and Lentivirus Infection

Taking RFWD3 and BIRC5 as templates, the corresponding RNAi target sequences and overexpression plasmids were designed by Shanghai Bioscienceres Co., Ltd. (Shanghai, China). The RFWD3 and BIRC5 target sequences were inserted into BR-V-108 vector through the restriction sites at both ends and subsequently transformed into TOP 10 E. coli competent cells (Tiangen, Beijing, China). The positive recombinants were screened by PCR. The EndoFree maxi plasmid kit (Tiangen, Beijing, China) was utilized to extract plasmid. A three-plasmid BR-V108, BR-V307, BR-V112 co-infection system was used to collect 239T cell supernatant at 48 h and 72 h after infection and the quality of lentivirus was evaluated. Finally, RKO or HCT-116 cells in logarithmic growth phase were infected by adding 20 μL 1 × 10^8^ TU/mL lentivirus, culturing in 1640 medium with 10% FBS in a 6-well dish with 2 × 10^5^ cells per well. The infection efficiency and knockdown efficiency were evaluated by fluorescence microscopy (micropublisher 3.3RTV, Olympus, Tokyo, Japan), qRT-PCR and western blot.

### RNA Extraction and qRT-PCR

The total RNA was isolated according to manufacturer’s protocol of the TRIzol reagent (Sigma, St. Louis, MO, United States), and the production was reversely transcribed to obtain cDNA using the Promega M-MLV Kit (Promega Corporation, Madison, Wisconsin, United States). The qRT-PCR experiment was performed on the platform of Applied Biosystems 7500 Real-Time PCR system (Cat #VII7, ABI Corporation). The qRT-PCR reaction volume was 10 μL according to SYBR Green Mastermixs Kit (Vazyme, Nanjing, Jiangsu, China). The thermal profile consisted of an initial denaturation at 95°C for 3 min followed by 40 cycles at 95°C for 3 s and at 60°C for 30 s. The relative expression of mRNA was calculated by the 2^–ΔΔCt^ method. The primers sequences (5′–3′Supplementary Table 2.

### Western Blot Assay and Co–immunoprecipitation (Co–IP)

After infection, RKO and HCT–116 were collected and lysed with 1 × Lysis Buffer lysis (Cell Signal Technology, Danvers, MA). At the same time, 10% SDS–PAGE was used to segregate the total proteins and transferred into PVDF membranes followed by blocking with a blocking solution (TBST solution containing 5% skim milk) at room temperature for 1 h. Next, the membranes were incubated with antibodies and washed with TBST solution for three times (10 min/time). Finally, the ECL + plus^TM^ Western blotting system kit (Amersham, Chicago, IL, United States) was used for color rendering and X-ray imaging was captured.

In Co-IP analysis, HCT-116 cells were lysed, and total proteins were extracted. Then, 1.0–1.2 mg proteins were incubated with antibody overnight, followed by 2 h of incubation with 20 μL beads at 4°C. After that, the complex was lysed and incubated at 95–100°C for 10 min. Then the proteins in the immunocomplex were separated by 10% SDS-PAGE for western blot assay. Finally, the corresponding primary and secondary antibodies were incubated to identify interacting proteins. Antibodies used in western blot assay were shown in Supplementary Table 1.

### Celigo Cell Counting Assay

RKO and HCT-116 cells were collected after infection. When the cells reached 70 to 90% confluence, they were then seeded into 96-well plates at the density of 2,000 cells/well, placing in an incubator with 5% CO_2_ at 37°C. The cell images were taken by Celigo image cytometer (Nexcelom Bioscience, Lawrence, MA, United States) and a continuous 5-day cell proliferation curve was drawn.

### Colony Formation Assay

Lentivirus-infected RKO and HCT-116 cells were collected, digested and resuspended (2,500 cells/mL). For colony formation, the cell suspension was seeded in a 6-well plate (2 mL/well). The cells were cultured for 8 days and the medium was changed every 3 days to form colony. Visible clones in 6-well plate were recorded by fluorescence microscope (micropublisher 3.3RTV, Olympus, Tokyo, Japan). Finally, the cells were washed with PBS, fixed with 1 mL 4% paraformaldehyde and stained by 500 μL Giemsa (Dingguo, Shanghai, China).

### Cell Migration Assay

The wound-healing assay was performed to evaluate cell migration ability. RKO and HCT-116 cells, infected with lentivirus, were seeded into a 96-well plate (5 × 10^4^ cells/well). Then, the cells were incubated in an incubator with 5% CO_2_ at 37°C, and were observed in a microscope at 48 h and 72 h. The experiment was repeated 3 times and the migration rate of cells was evaluated based on the scratch images.

For the transwell assay, RKO and HCT-116 cells with lentivirus were cultured to reach the density of 1 × 10^5^ cells/mL and loaded into the upper chamber containing serum-free medium. Then, the upper chamber was transferred to the lower chamber with 30% FBS and incubated for 72 h. Finally, 400 μL Giemsa was added for cell staining and the cell migration ability was quantified.

### Cell Apoptosis Assay

Lentivirus-infected RKO and HCT-116 cells were cultured in 6-well plates (2 mL/well) for 5 days. 10 μL Annexin V-APC (eBioscience, Thermo Fisher) was added for 10–15 min at room temperature in the dark to stain. The cell apoptosis level was measured using FACSCalibur (BD Biosciences, San Jose, CA, United States).

### Human Phosphorylation Array

The Human Phosphorylation Array was performed to explore the effects of loss of RFWD3-function on the phosphorylation-related proteins in RKO cells. After the cells were lysed, the Handling Array membranes were blocked using 2 mL 1 × Wash Buffer II and incubated with cell lysates and 1 × Biotin-conjugated Anti-Cytokines overnight at 4°C. Membrane intensity was acquired *via* ECL and pixel densities were analyzed using ImageJ software version 7.0 [US National Institutes of Health (NIH), Bethesda, MD, United States].

### PrimeView Human Gene Expression Array

Gene expression in HCT-116 cells was detected with microarrays in Shanghai Bioscienceres, Co., Ltd. (Shanghai, China). Briefly, the total RNA was extracted by the RNeasy kit (Sigma, St. Louis, MO, United States), the quality and integrity of which were determined by Nanodrop 2000 spectrometer (Thermo, Waltham, MA, United States) and Agilent 2100 and Agilent RNA 6000 Nano Kit (Agilent, Santa Clara, CA, United States). According to the manufacturer’s instruction, the RNA sequencing was performed with Affymetrix human GeneChip PrimeView and the outcomes were scanned by Affymetrix Scanner 3000 (Affymetrix, Santa Clara, CA, United States). The statistical significance of raw data was completed by a Welch *t*-test with Benjamini-Hochberg FDR (| Fold Change| ≥ 2 and *FDR* < 0.05 as significant). Significant difference analysis and functional analysis based on Ingenuity Pathway Analysis (IPA) (Qiagen, Hilden, Germany) was performed, and | Z - score| > 2 is considered valuable.

### Dual-Luciferase Assay

The BIRC5 promoter region fragment was amplified and cloned into the luciferase reporter vector GL002 (Promega Madison, United States), named GL002-BIRC5. The mutant construct GL002-BIRC5-MUT was produced by site-directed mutagenesis, and GL002-BIRC5-WT was used as a negative control. The firefly luciferase value and the Renilla luciferase signal were measured using the Promega dual luciferase system (Cat. No. E2940, Madison, United States). Each experimental analysis was repeated 3 times.

### Chromatin Immunoprecipitation (ChIP)-qPCR Assay

HCT-116 cells overexpressing RFWD3 were cross-linked with formaldehyde, lysed in SDS buffer, and mechanically sheared by sonication to fragment DNA. Protein–DNA complexes were precipitated using 2 μg control normal mouse IgG (Sigma, Cat. No. I5381), 2 μg Histone H3 (D2B12) XP^®^ Rabbit mAb (CST, Cat. No.4620) and 4 μg anti-E2F1 (Proteintech, Cat. No. 66515-1-Ig) antibody. After that, the eluted DNA fragment was detected with the primers specific for BIRC5 promoter and SYBR premix (Vazyme).

### The Construction of Nude Mouse Tumor Formation Model

The study was approved by Laboratory Animal Welfare and Ethics Committee of Third Military Medical University. RKO cells with or without RFWD3 were subcutaneously injected into the four-week-old female BALB-c nude mice from Shanghai Weitong Lihua Animal Research Co., Ltd. (Shanghai, China) to construct xenograft models (10 mice/group). L and W of tumors were recorded to obtain tumor volume (L represent longest dimension and W means dimension perpendicular to length, and tumor volume was calculated as π/6 × L × W2). 0.7% sodium pentobarbital was injected intraperitoneally for several min on the last day of feeding, and the fluorescence was observed by the *in vivo* imaging system (IVIS Spectrum, Perkin Elmer). After 43 days, the mice were sacrificed and the tumors were removed to weigh and photograph, and finally frozen in liquid nitrogen and stored at -80°C.

### Ki-67 Staining

First, the tumor tissues were fixed by 4% paraformaldehyde and 0.3% TritonX-100. Then, the 5 μm sections were embedded with paraffin for IHC staining. Next, the slides were incubated with primary antibody Ki-67 at 4°C overnight and the secondary antibody was added as above methods (Supplementary Table 1). Finally, the slides were stained with Hematoxylin and Eosin (Baso, Zhuhai, Guangdong, China), and observed under an inverted microscope (IX73, Olympus, Tokyo, Japan).

### Statistical Analysis

All data were analyzed using GraphPad Prism 8 (San Diego, CA, United States) and SPSS 19.0 (IBM, SPSS, Chicago, IL, United States), and presented as the mean ± SD. Student’s *t*-test and one-way ANOVA were used to analyze the statistical significance. The Mann-Whitney U analysis were used to assess the relationship between RFWD3 expression and characteristics of CRC patients. *P* < 0.05 was considered to be significantly different. All the experiments were conducted in triplicate.

## Results

### RFWD3 Was Highly Expressed and Associated With Poor Prognosis in Colorectal Tumor

**FIGURE 1:**
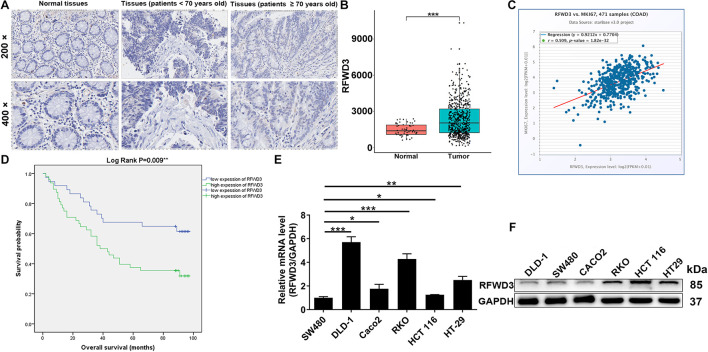
RFWD3 was up-regulated in colorectal cancer tissues and cell lines. **(A)** The expression levels of RFWD3 in colorectal cancer tumor tissues and para-carcinoma tissues were determined by immunohistochemical staining. **(B)** Data mining of TCGA database showed that expression of RFWD3 was relatively higher in colorectal cancer tissues compared with normal tissues. **(C)** A positive correlation between RFWD3 level and Ki-67 expression was observed in ENCORI Pan-Cancer Analysis Platform. **(D)** Kaplan-Meier survival analysis was performed to reveal the relationship between RFWD3 expression and prognosis of colorectal cancer patients. **(E,F)** The mRNA and protein expression of RFWD3 in colorectal cancer cell lines was evaluated by qRT-PCR **(E)** and western blot **(F)**. Results were presented as mean ± SD. * *P* < 0.05, ** *P* < 0.01, *** *P* < 0.001.

The differential RFWD3 expression within colorectal tumor tissues and adjacent normal tissues was investigated using immunohistochemistry analysis. We noticed that, from the immunohistochemistry analysis, RFWD3 expression was upregulated in 48 cases of tumor tissues, but only in 4 cases of normal tissues (Figure 1A and Table 1). Meanwhile, RFWD3 level was upregulated in CRC tissues based on the results obtained from the RNA-seq data collected from TCGA database (*P* < 0.001, Figure 1B). Moreover, we observed a positive correlation between RFWD3 level and Ki-67 expression in ENCORI Pan-Cancer Analysis Platform (Figure 1C). Subsequent correlation analysis based on RFWD3 expression and pathological characteristics of CRC patients revealed its significant relationship with patients’ age (Table 2). Of note, the Kaplan-Meier survival analysis demonstrated that higher expression of RFWD3 was significantly linked to poorer prognosis of CRC patients, as well as shorter survival period (Figure 1D). Except for detecting RFWD3 level in CRC tumor tissues, the mRNA and protein expression of RFWD3 in human colorectal adenocarcinoma epithelial cell DLD-1 and a panel of CRC cell lines was evaluated by qRT-PCR and western blot analysis. As presented in Figures 1E,F, despite ubiquitously upregulated RFWD3, we selected RKO and HCT 116 cells, with relatively higher RFWD3 protein expression, for further studies. Altogether, these experimental data and bioinformatics showed potential involvement of RFWD3 in the development and progression of CRC.

**TABLE 1:** Expression patterns of RFWD3 in colorectal cancer tissues and para-carcinoma tissues revealed in immunohistochemistry analysis.

**RFWD3 expression**	**Tumor tissue**		**Para-carcinoma tissue**		***P* value**
	**Cases**	**Percentage**	**Cases**	**Percentage**	
Low	37	43.5%	57	93.4%	< 0.001
High	48	56.5%	4	6.6%	

**TABLE 2:** Relationship between RFWD3 expression and tumor characteristics in patients with colorectal cancer.

**Features**	**No. of patients**	**RFWD3 expression**		***P* value**
		**low**	**high**	
All patients	85	37	48	
Age (years)				0.013
<70	42	24	18	
≥ 70	43	13	30	
Gender				0.947
Male	44	19	25	
Female	41	18	23	
Tumor size				0.757
<5cm	37	17	20	
≥ 5cm	47	20	27	
Grade				0.658
I	1	0	1	
II	70	32	38	
III	13	5	8	
IV	1	0	1	
Stage				0.229
1	6	3	3	
2	43	21	22	
3	35	13	22	
4	1	0	1	
T				0.212
T1	1	1	0	
T2	5	2	3	
T3	62	29	33	
T4	17	5	12	
(N)				0.285
N0	49	24	25	
N1	26	9	17	
N2	10	4	6	
Lymph node positive				0.177
=0	47	24	23	
>0	36	13	23	

### Silencing RFWD3 Suppressed CRC Cell Growth and Migration *in vitro*

**FIGURE 2:**
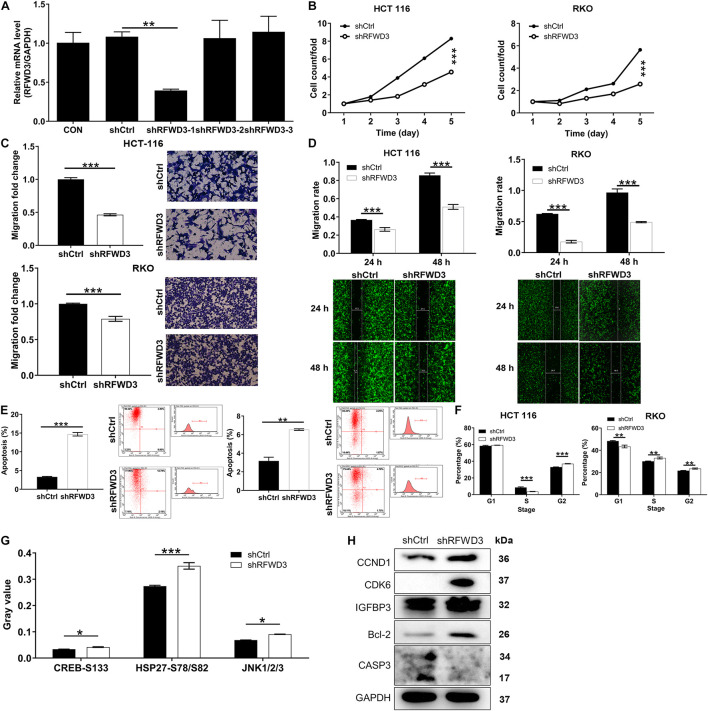
RFWD3 knockdown inhibited colorectal cancer development *in vitro***. (A)** The knockdown efficiencies of shRFWD3-1, shRFWD3-2, and shRFWD3-3 were detected by qRT-PCR. **(B)** The cell proliferation rate was evaluated in colorectal cancer cell lines after infection by Celigo cell counting assay. **(C,D)** The migration rate of cells was detected in colorectal cancer cell lines after infection by transwell assay **(C)** and wound-healing assay **(D)**. Magnification times: 200×. **(E,F)** The effects of RFWD3 knockdown on cell apoptosis **(E)** and cell cycle **(F)** were examined by flow cytometry. **(G)** The levels of phosphorylation-related proteins in RKO cells infected with shCtrl and shRFWD3 were measured using a human phosphorylation array. Protein expression was presented in gray value. **(H)** The expression of CCND1, CDK6, Bcl-2, IGFBP3, and CASP3 was detected by western blot. Results were presented as mean ± SD. * *P* < 0.05, ** *P* < 0.01, *** *P* < 0.001.

The above observations prompted us to evaluate the impacts of RFWD3 expression on CRC *in vitro*. Intending to determine if RFWD3 disruption elicited the changes of CRC cell phenotype, we first infected HCT-116 cells with three lentivirus plasmid shRFWD3-1, shRFWD3-2 and shRFWD3-3. Immediately afterward, shRFWD3-1 achieved optimum knockdown efficiency and was employed for next study (*P* < 0.01, Figure 2A). The infection/knockdown efficiency of shRFWD3-1 in RKO and HCT 116 cells was evaluated by observing fluorescence inside cells, qRT-PCR and western blot analysis, which validated downregulation of RFWD3 in the presence of shRFWD3-1 (Supplementary Figure 1). Based on assessment on the malignant phenotypes of CRC cells by Celigo-based method, wound-healing/transwell migration assay, RFWD3 downregulation suppressed cell viability and migration (*P* < 0.001, Figures 2B–D). Moreover, we noticed that both CRC cells with RFWD3 disruption enhanced apoptosis (Figure 2E) and appeared a phenomenon of cell cycle arrest (Figure 2F). In order to preliminarily study the potential mechanism of RFWD3’s action, we analyzed changes in the levels of 37 phosphorylation-related proteins using a human phosphorylation array in shCtrl and shRFWD3 RKO cells. Following this was the upregulation of CREB-S133, HSP27-S78/S82, and JNK1/2/3 (Figure 2G and Supplementary Figures 2A,B). Next, western blot analysis was performed on RFWD3-silenced RKO cells, indicating the upregulation of CCND1, CDK6, Bcl-2, and IGFBP3 (Figure 2H). Considering that RFWD3 depletion might be toxic to most cell lines, we also infected human normal colorectal mucosal cells FHC with shCtrl and shRFWD3 and detected the alterations of cell proliferation. The results indicated that knocking down RFWD3 did not significantly affect cell proliferation of FHC cells (Supplementary Figure 2C). Taken together, we concluded that RFWD3 could promote the development of CRC *in vitro*.

### Inhibiting RFWD3 Impaired CRC Tumorigenesis *in vivo*

**FIGURE 3:**
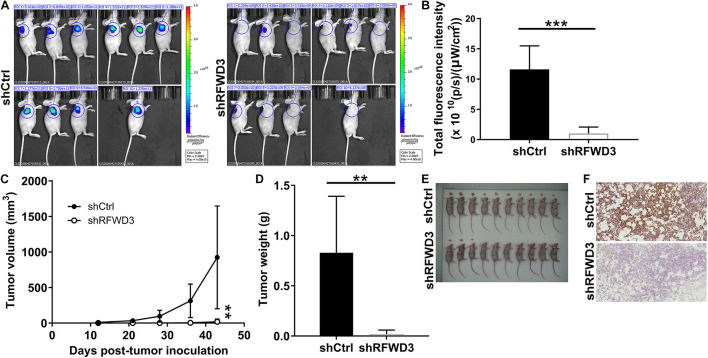
RFWD3 knockdown inhibited colorectal cancer tumor growth *in vivo*. **(A)** A nude mice model of RFWD3 knockdown was constructed. **(B)** The fluorescence intensity was obtained through injection of D-Luciferase before sacrificing the mice. **(C)** L and W of tumors were recorded to obtain tumor volume (L represent longest dimension and W means dimension perpendicular to length, and tumor volume was calculated as π/6 × L × W2). **(D)** The weight of tumors was measured after sacrificing mice. **(E)** The photograph of tumors was taken after removing tumors. **(F)** The level of Ki-67 was detected by IHC in tumor sections from mice. Results were presented as mean ± SD. ** *P* < 0.01, *** *P* < 0.001.

Next, we used well-established xenograft mouse models to unveil key functions of RFWD3 depletion in CRC progression *in vivo* (Figure 3A). During the xenografting, such reduction of fluorescence in the shRFWD3 group was thought to be impaired in the growth of tumors formed by RKO cells with shRFWD3 (*P* < 0.001, Figure 3B). The mice were sacrificed 43 days after the xenografting. In consistent with the result of *in vivo* imaging, the observations that tumor from mice injected RFWD3-silenced RKO cells displayed decreased volume and weight suggested a possible contribution of RFWD3 depletion in impairing tumorigenesis in these mice (Figures 3C–E). Coherently, the loss of RFWD3 function suppressed Ki-67 level in tumor tissues from shRFWD3 mice, as reflected by Figure 3F. Therefore, the findings in this context were in agreement with the above *in vitro* studies, indicating that RFWD3 knockdown restricted CRC tumorigenesis *in vivo*.

### RFWD3 Regulates BIRC5 Level *via* E2F1 Transcription

**FIGURE 4:**
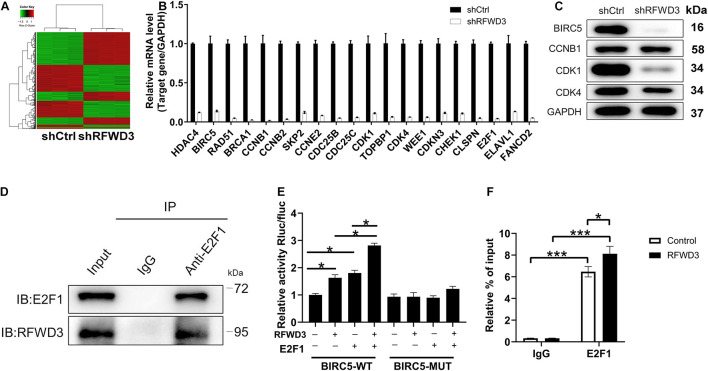
Exploration and verification of underlying mechanism of RFWD3 regulating colorectal cancer. **(A)** The heatmap of DEGs was identified by Affymetrix Human Gene Chip Prime View analysis in HCT 116 cells infected with shCtrl (*n* = 3) or shRFWD3 (*n* = 3). **(B,C)** The expression of several most significantly DEGs was determined by qRT-PCR **(B)** and western blot **(C)** in HCT 116 cells following infection. **(D)** Co-IP assay was used to verify whether there was protein interaction between RFWD3 and E2F1. **(E)** The dual-luciferase assay was conducted to explore whether RFWD3 regulated BIRC5 promoter activity. **(F)** ChIP-qPCR assay was conducted to confirm that E2F1 bound to the CDK1 promoter. The data are expressed as mean ± SD. * *P* < 0.05, *** *P* < 0.001.

In this section, we wanted to dissect the underlying mechanism of how RFWD3 promotes CRC. Therefore, we performed Affymetrix Human Gene Chip Prime View analysis on HCT 116 cells. Of the 1305 differentially expressed genes (DEGs) found in shRFWD3 and shCtrl HCT 116 cells, 629 were up-regulated and 676 were down-regulated based on the threshold of absolute fold change ≥ 2 and *FDR* < 0.05 (Figure 4A and Supplementary Figure 3A). Hereafter, all DEGs were enriched in the canonical signaling pathway or IPA disease and function, indicating that the PPAR Signaling, Cyclins and Cell Cycle Regulation, Superpathway of Cholesterol Biosynthesis and Estrogen-mediated S-phase Entry were significantly restrained (Supplementary Figures 3B,C). Furthermore, the DEGs with the highest fold change were further screened by qRT-PCR detection and western blot analysis. Especially, BIRC5 mRNA and protein expression levels were found to be apparently decreased in RFWD3-silenced HCT 116 cells (Figures 4B,C). IHC analysis confirmed, in keeping with RFWD3 expression in CRC tissues, a trend toward upregulation of BIRC5 (Supplementary Figure 3D). We also performed correlation analysis between RFWD3 and BIRC5 expression. As expected, BIRC5 expression was positively associated with RFWD3 level (Supplementary Figure 3E). Accordingly, we speculated that BIRC5 might be a downstream gene of RFWD3 regulating CRC. Subsequently, through analyzing the TRRUST website,^1^ we predicted that the upstream transcription factor of BIRC5 was E2F1. Combining with the genecards website,^2^ we found that both E2F1 and RFWD3 are located in the nucleus, and further proposed that there is an interaction between E2F1 and RFWD3, which was evidenced by additional Co-IP assay, implying that RFWD3 could directly bound to E2F1 in HCT-116 cells (Figure 4D). From this, we inferred that RFWD3 may regulate the expression of BIRC5 through E2F1. We thus generated a luciferase reporter construct with wild-type (WT) and E2F1 binding motif-mutated BIRC5 promoter (MUT) to conduct dual-luciferase assay. The results indicated that RFWD3 overexpression significantly increased BIRC5 promoter driven luciferase activity in the WT group but not the MUT group. More importantly, RFWD3 overexpression promoted the binding of E2F1 to the BIRC5 promoter (Figure 4E). A ChIP-qPCR assay confirmed that RFWD3 overexpression enhanced the transcriptional regulation of BIRC5 by E2F1 (Figure 4F). Collectively, our findings demonstrated that RFWD3 could transcriptionally activate BIRC5 by interacting with E2F1 in HCT-116 cells.

### Silencing RFWD3 Restricted CRC Cell Growth and Mobility *via* Downregulating BIRC5 Expression

**FIGURE 5:**
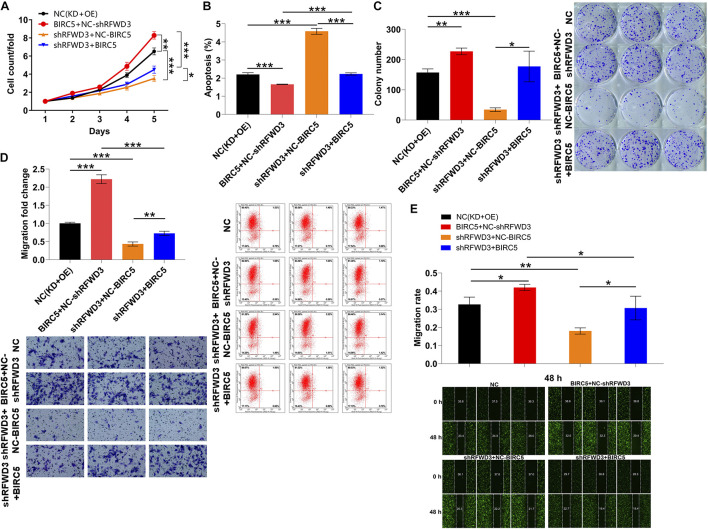
Overexpression of BIRC5 alleviates the inhibitory effects of RFWD3 knockdown on colorectal cancer cells. **(A–C)** RKO cell models were subjected to the detection of cell proliferation **(A)**, cell apoptosis **(B)** and colony formation **(C)**. The alterations of cell migration were tested *via* transwell assay **(D)** and wound-healing assay **(E)**. NC (OE + KD) as negative control; BIRC5 + NC-shRFWD3 as overexpressed BIRC5; shRFWD3 + NC-BIRC5 as RFWD3 downregulation; BIRC5 + shRFWD3 as simultaneously downregulated RFWD3 and overexpressed BIRC5. The data are expressed as mean ± SD. * *P* < 0.05, ** *P* < 0.01, *** *P* < 0.001.

To this end, in order to further confirm the role played by BIRC5 in RFWD3-mediated CRC, we constructed BIRC5-overexpressing, RFWD3-knocking down and RFWD3-knocking down combining with BIRC5-overexpressing RKO cell models to perform *in vitro* experiments. The data shown in Supplementary Figure 4 suggested that the above cell models were established smoothly. Besides, we applied the Celigo-based method, wound-healing/transwell migration assay and flow cytometry experiments to observe the alterations of cell phenotypes in these RKO cell models and obtained the following data. In line with the previous data, silencing RFWD3 significantly suppressed cell proliferation and migration. On the contrary, overexpressing BIRC5 enhanced RKO cell proliferation, migration and colony formation, simultaneously attenuated apoptosis. More importantly, upon overexpressing BIRC5 in RKO cells with down-regulated RFWD3, the inhibitory effects of cell proliferation, migration and colony formation could be reversed, while the capacity of cell apoptosis was ameliorated, demonstrating the effects of RFWD3 depletion was mainly due to BIRC5 suppression (Figures 5A–E). On the other hand, we also stably infected RKO cells with RFWD3, shBIRC5 and shBIRC5 + RFWD3 lentiviruses to merely over-express RFWD3, merely down-regulate BIRC5, and simultaneously down-regulate BIRC5 and over-express RFWD3, respectively (Supplementary Figure 5). We found that cell proliferation, migration and colony formation were apparently promoted by RFWD3 overexpression, while were notably cut down by the down-regulation of BIRC5. Moreover, flow cytometry assay reflected that the decreased apoptosis caused by RFWD3 overexpression could be increased *via* BIRC5 knockdown (Supplementary Figure 6). As per the results presented so far, we concluded that the promotion effects of RFWD3 on cell proliferation and migration of CRC cells could be rescued *via* down-regulating BIRC5.

## Discussion

Colorectal cancer (CRC), as the third most common disease worldwide, occupies a large proportion of cancer-related death (Xie et al., 2020). Despite some obvious improvement in the survival rate of CRC patients, it is still significantly lower than that of other cancer patients (Zhang et al., 2016). Chemotherapy and radiotherapy are currently usual methods of treatment for CRC patients (Mcquade et al., 2017). However, these therapeutic options often have no effects for patients in advanced stage or distant metastasis CRC. Targeted therapy is a promising treatment regimen for improving outcomes of patients with advanced CRC. Mounting studies have reported that tumor-associated genes are aberrantly expressed and linked to the initiation and development of CRC (Tan et al., 2020; Xu et al., 2020). Thus, exploring the biological function taken by these genes in CRC can be useful for finding novel biomarkers and understanding the mechanism of CRC progression.

RFWD3, also called RNF201 and FLJ10520, was initially identified as a substrate of ATM/ATR from a proteomic screen (Liu et al., 2011). Subsequent studies demonstrated that RFWD3 is an E3 ubiquitin ligase that positively regulates p53 levels in response to DNA damage (Fu et al., 2010). The published literature mentioned that the high expression of RFWD3 was involved in the occurrence and development of gastric carcinoma, and may be an important poor prognostic factor of gastric carcinoma (Jia et al., 2020). Our present study, through IHC analysis and TCGA database, indicated that RFWD3 expression was significantly higher in CRC tissues compared with matched para-tumorous tissues. Besides, RFWD3 mRNA levels were significantly increased among CRC cell lines. So as to reveal the relationship between high expression of RFWD3 with CRC cell phenotypes, we down-regulated RFWD3 expression in both RKO and HCT 116 cells. We found that downregulation of RFWD3 expression promoted CRC cell apoptosis, suppressed cell proliferation and migration as well as arrested cell cycle. The resulting *in vitro* findings showed that down-regulation of RFWD3 expression suppressed CRC development, which was also verified by the data obtained in mice. At the mechanism level, we found that the transcription level of BIRC5 was diminished upon knocking down RFWD3. More importantly, BIRC5 was strongly expressed in CRC and associated with RFWD3 level. Accordingly, we initially identified BIRC5 as a downstream gene of RFWD3 regulating CRC.

The baculoviral inhibitor of apoptosis repeat containing-5 (BIRC5), also known as survivin, is the smallest member of inhibitor of apoptosis (IAP) family of proteins with anti-apoptotic, as well as, mitotic regulatory functions. BIRC5 inhibits apoptosis by interfering with the function of caspase-3, caspase-7, and caspase-9 and also act in caspase-independent pathways (Lv et al., 2010). In the past few years, BIRC5 has been extensively used as a biomarker for early prediction of human malignancies (Lo Muzio et al., 2003). It has been reported that BIRC5 was overexpression in more than half of breast cancer patients (Yie et al., 2006). In addition to breast cancer, BIRC5 expression has been associated with prognosis and diagnosis of multiple other human cancers such as acute lymphoblastic leukemia (Ahmed et al., 2012), prolactinoma (Dellal et al., 2015), pancreatic cancer (Dong et al., 2015) and non-small cell lung cancer (Chen et al., 2010). On the other hand, BIRC5 overexpression is related to adverse outcome in breast, lung, colorectal, prostate, and ovarian cancer and is thought to be a promising cancer diagnostic biomarker (Duffy et al., 2007; Fawzy et al., 2012). There has been also many studies showing that urine BIRC5 level can be used as a diagnostic indicator of bladder cancer (Weikert et al., 2010) and lung cancer (Wu et al., 2009). Recently, serum BIRC5 levels has been found to be positively linked to early diagnosis in majority of human cancers (Gunaldi et al., 2018). Here, we proposed how BIRC5 is involved in RFWD3-mediated CRC progression. Subsequently, we found that E2F1 is an upstream transcription factor of BIRC5. Notably, there is a protein interaction between E2F1 and RFWD3. As a transcription factor, E2F1 is involved in a variety of biological processes such as cell cycle regulation and apoptosis. In addition, E2F1 regulates the expression of various cytokines and growth factor receptors to establish a feedback mechanism (Ertosun et al., 2016). A recent research from Lu et al. revealed that E2F1 transcriptionally regulated cell cycle regulator cyclin A2 (CCNA2) expression to promote tumorigenicity of TNBC (Lu et al., 2021). Here, further study based on a dual-luciferase assay and a ChIP-qPCR assay revealed that RFWD3 could facilitate the binding of E2F1 to the BIRC5 promoter and transcriptionally activate BIRC5 by interacting with E2F1 in HCT-116 cells. Additional loss- and gain- of function experiments demonstrated that overexpression of BIRC5 alleviated the inhibitory effects of RFWD3 knockdown in CRC cells. In conclusion, our study highlighted RFWD3 may function as a tumor promotor in CRC *via* E2F1 transcriptional regulation of BIRC5, which might be used as a future therapeutic target for the more effective treatment of CRC.

## Data Availability Statement

Publicly available datasets were analyzed in this study. This data can be found here: https://www.cancer.gov/about-nci/organization/ccg/research/structural-genomics/tcga. In addition, we have uploaded gene expression array data to Gene Expression Omnibus (GEO) and the accession number(s) is GSE185122.

## Ethics Statement

The studies involving human participants were reviewed and approved by ethical approval was obtained from the Ethics Committee of Third Military Medical University. The patients/participants provided their written informed consent to participate in this study. The animal study was reviewed and approved by all animal experiments conformed to the European Parliament Directive (2010/63/EU) and were approved by the Laboratory Animal Welfare and Ethics Committee of Third Military Medical University.

## Author Contributions

DC and YW designed this research. FX, YT, and MC operated the cell and animal experiments. ZX, LF, and GR conducted the data procession and analysis. FX and YC completed the manuscript which was reviewed by DC and YW. All the authors have confirmed the submission of this manuscript.

## Conflict of Interest

The authors declare that the research was conducted in the absence of any commercial or financial relationships that could be construed as a potential conflict of interest.

## Publisher’s Note

All claims expressed in this article are solely those of the authors and do not necessarily represent those of their affiliated organizations, or those of the publisher, the editors and the reviewers. Any product that may be evaluated in this article, or claim that may be made by its manufacturer, is not guaranteed or endorsed by the publisher.

## Footnotes

1
https://www.grnpedia.org/trrust/result_tonly.php?gene=BIRC5&species=human&confirm=

2
https://www.genecards.org/
