# Supplementary material for: RFWD3 Participates in the Occurrence and Development of Colorectal Cancer via E2F1 Transcriptional Regulation of BIRC5
Source: Front Cell Dev Biol. 2021 Oct 12;9:675356. doi: 10.3389/fcell.2021.675356 (PMC8547426; doi:10.3389/fcell.2021.675356)
Supplement: Supplementary file 1 [file Data_Sheet_1.pdf]

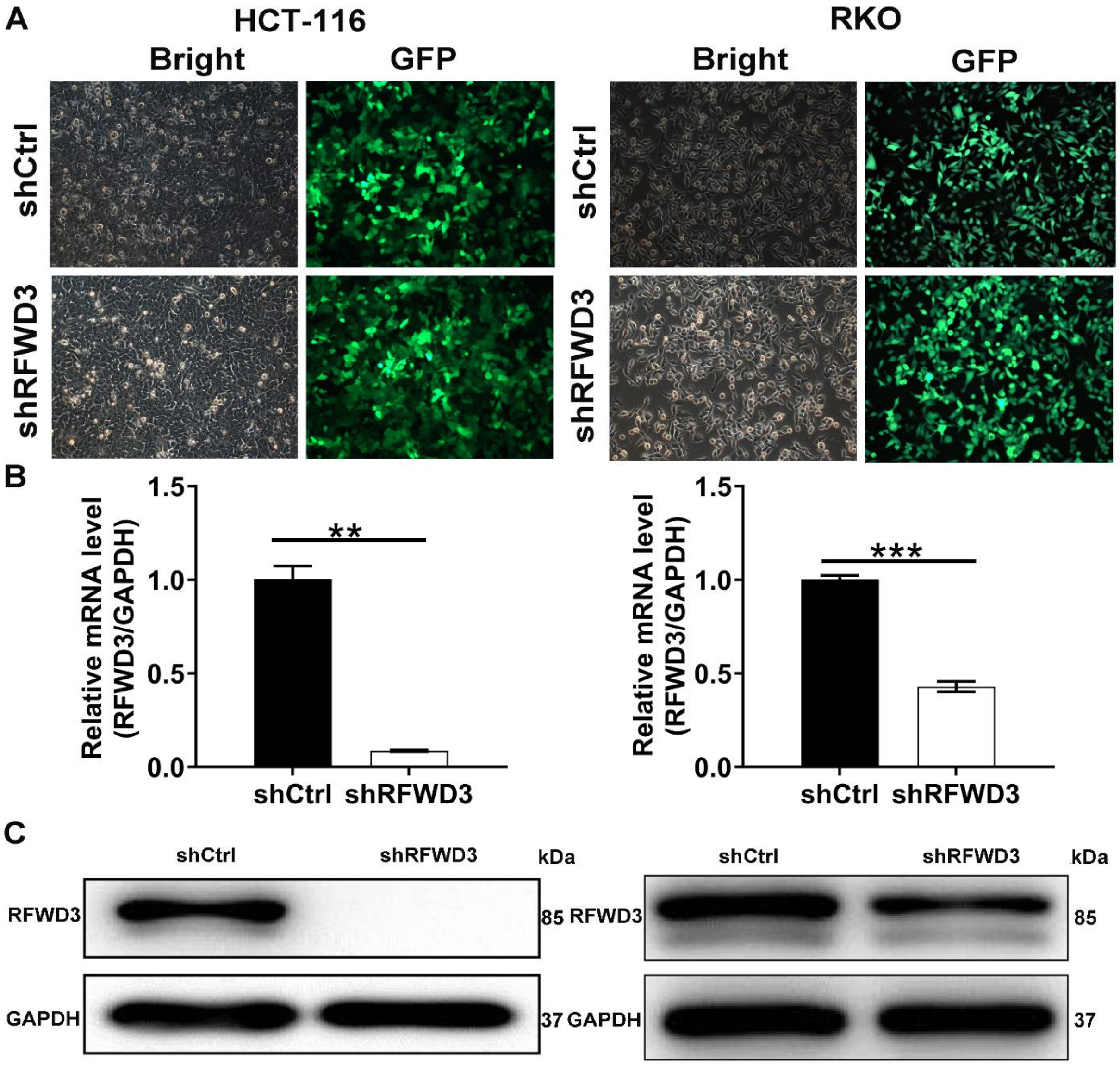

Figure S1. (A) The infection efficiencies of shRFWD3-1 in HCT 116 and RKO cells were evaluated through observing the fluorescence inside cells. Magnification times: 200 ×. (B) The RFWD3 expression in colorectal cancer cell lines after infection was analyzed by qRT-PCR. (C) The expression of RFWD3 protein in colorectal cancer cell lines after infection was detected by western blot.

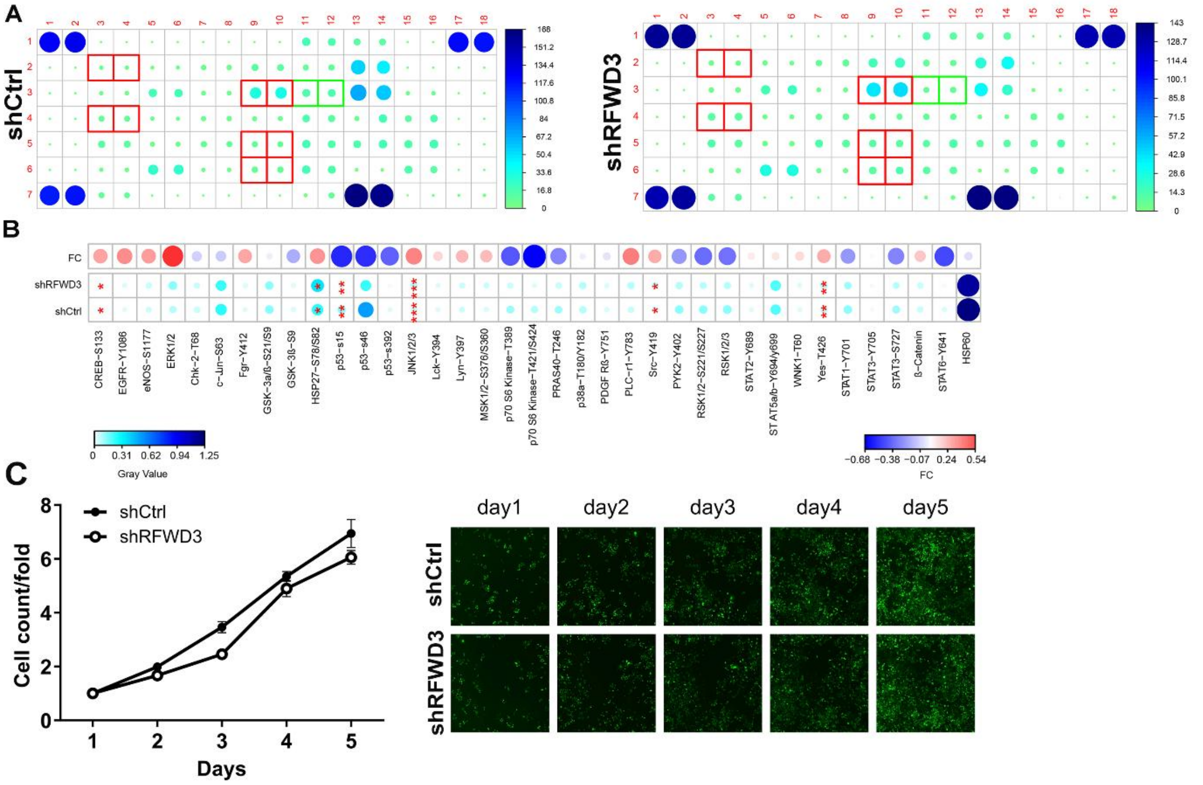

Figure S2. (A, B) The levels of phosphorylation-related proteins in RKO cells infected with shCtrl and shRFWD3 were measured using a human phosphorylation array. Protein expression was visualized by R studio. (C) The cell proliferation of FHC cells after infecting shCtrl and shRFWD3 was evaluated via Celigo cell counting assay. Results were presented as mean ± SD.

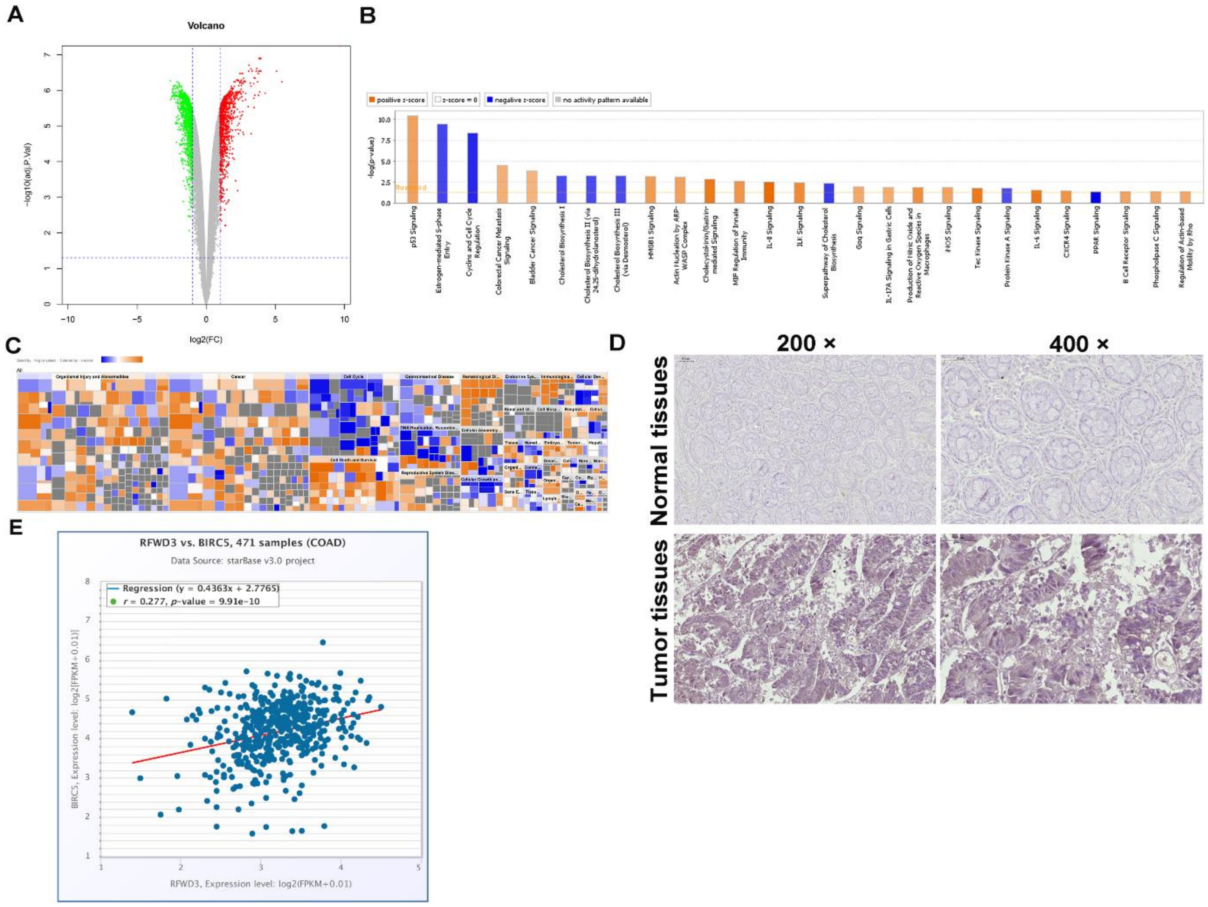

Figure S3. (A) The volcano plot of gene expression profiling in HCT 116 cells with or without RFWD3 knockdown. Red dots represent the upregulated DEGs, green dots represent the downregulated DEGs. (B, C) The enrichment of the DEGs in canonical signaling pathways (B) and IPA disease and function (C) was analyzed by IPA. (D) The expression levels of BIRC5 in colorectal cancer tumor tissues and para-carcinoma tissues were determined by immunohistochemical staining. (E) A positive correlation between RFWD3 level and BIRC5 expression was observed in ENCORI Pan-Cancer Analysis Platform.

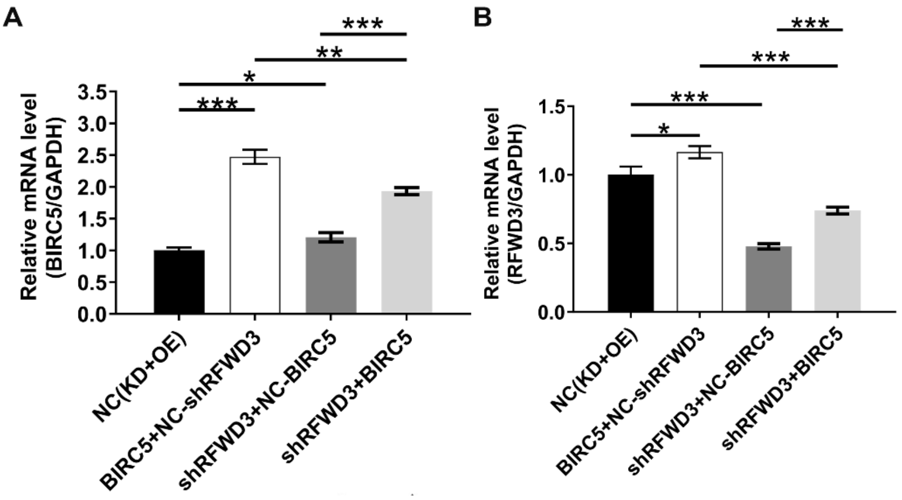

Figure S4. The mRNA levels of BIRC5 (A) and RFWD3 (B) were detected in RKO cell models after infection. NC (OE+KD) as negative control; BIRC5+NC-shRFWD3 as overexpressed BIRC5; shRFWD3+NC-BIRC5 as RFWD3 downregulation; BIRC5+shRFWD3 as simultaneously downregulated RFWD3 and overexpressed BIRC5. * $P < 0.05$, ** $P < 0.01$, *** $P < 0.001$.

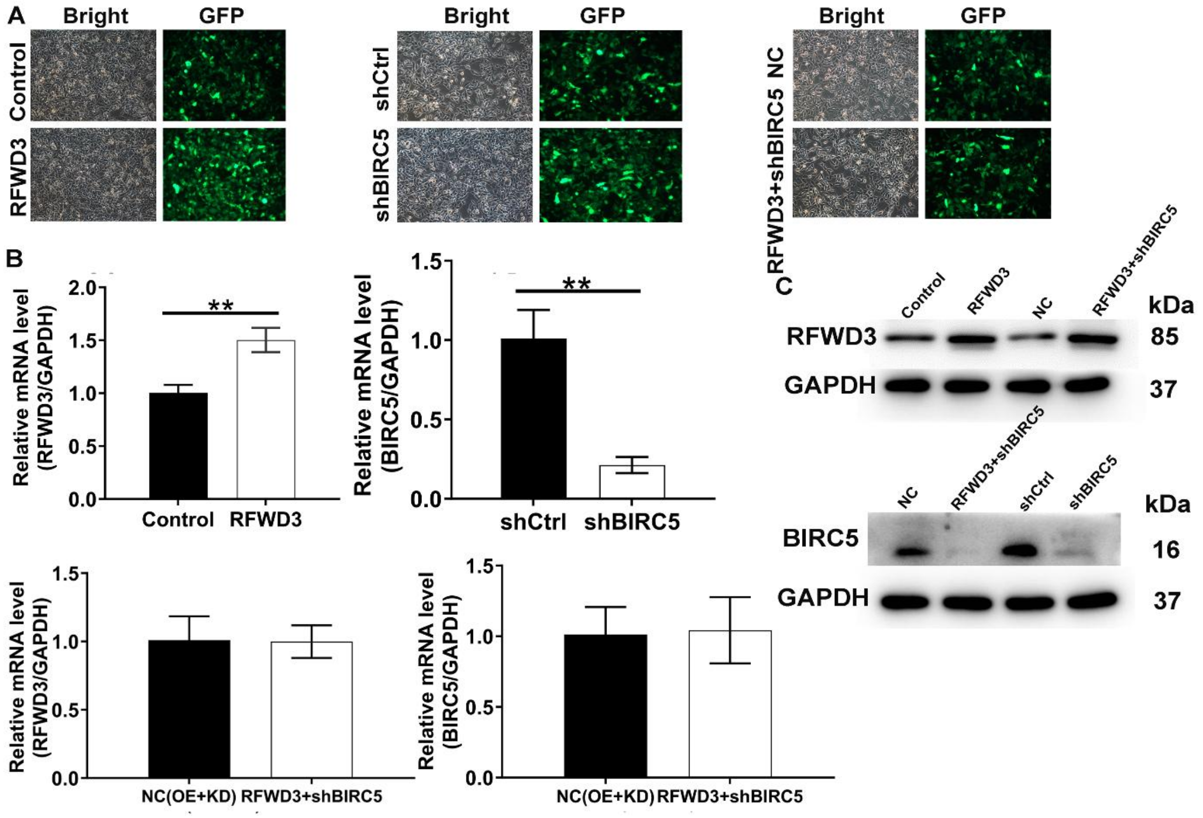

Figure S5. (A) The infection efficiencies of RFWD3, shBIRC5 and shBIRC5+ RFWD3 in RKO cells were evaluated through observing the fluorescence inside cells. Magnification times: 200×. (B) The RFWD3 and BIRC5 mRNA expression in RKO cell lines after infecting RFWD3, shBIRC5 and shBIRC5+ RFWD3 was analyzed by qRT-PCR. (C) The RFWD3 and BIRC5 protein expression in RKO cell lines after infecting RFWD3, shBIRC5 and shBIRC5+ RFWD3 was analyzed by western blot. ** $P < 0.01$.

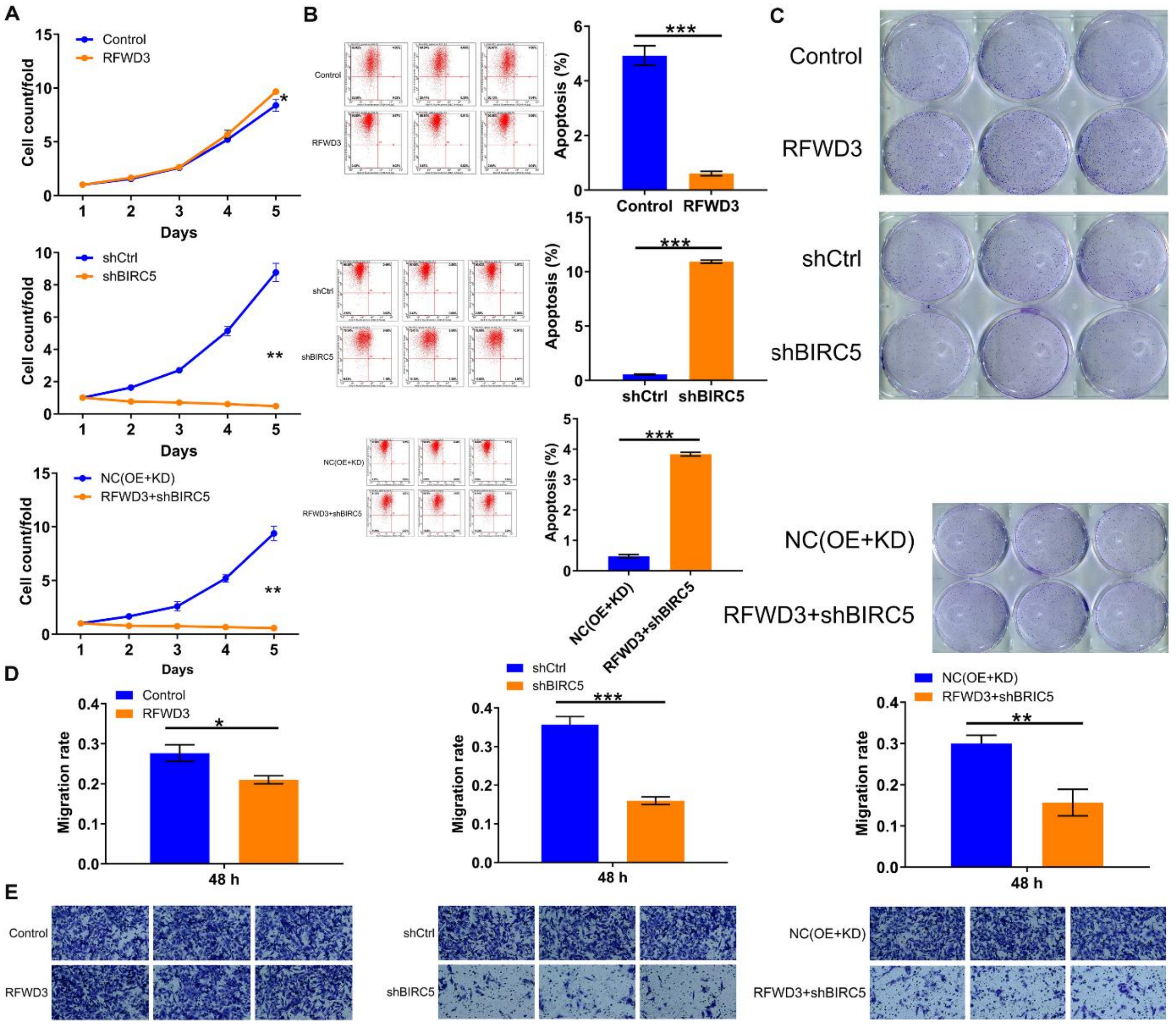

Figure S6. (A, B) Celigo cell counting assay (A) and the flow cytometry (B) were employed to assess the changes in cell proliferation and cell apoptosis in RFWD3, shBIRC5 and shBIRC5+ RFWD3 groups. (C) Colony formation assay was used to evaluate the ability of RKO cells to form colonies in RFWD3, shBIRC5 and shBIRC5+ RFWD3 groups. (D, E) The migration rate of cells was detected in RFWD3, shBIRC5 and shBIRC5+ RFWD3 groups by wound-healing assay (D) and transwell assay (E). The data are expressed as mean ± SD. * P < 0.05, ** P < 0.01, *** P < 0.001.

Supplementary Table 1. Antibodies used in western blotting and IHC.

Primary antibodies	Dilution in WB	Source species	Company	Catalog No.
RFWD3	1:500	Rabbit	biorbyt	orb304552
CDK6	1:1000	Rabbit	abcam	ab151247
CCND1	1:2000	Rabbit	abcam	ab134175
IGFBP3	1:500	Rabbit	Wuhan Sanying	10189-2-AP
Bcl-2	1:500	Mouse	santa cruz	sc-7382
CASP3	1:1000	Rabbit	abcam	ab13847
BIRC5	1:1000	Rabbit	abcam	ab469
CCNB1	1:3000	Rabbit	abcam	ab32053
CDK1	1:1500	Rabbit	abcam	ab133327
CDK4	1:1000	Rabbit	abcam	ab108357
E2F1	1:50/1:2000	Mouse	Proteintech	66515-1-Ig
GAPDH	1:3000	Rabbit	Bioworld	AP0063
DYKDDDDK Tag	1:50/1:1000	Rabbit	CST	14793
Secondary antibody	Dilution		Company	Catalog No.
Goat Anti-Rabbit	1:3000		Beyotime	A0208
Goat Anti-Mouse	1:3000		Beyotime	A0216

Primary antibodies	Dilution in IHC	Source species	Company	Catalog No.
RFWD3	1:100	Rabbit	biorbyt	orb304552
BIRC5	1:200	Rabbit	abcam	ab469
Ki67	1:400	Rabbit	abcam	ab6721
Secondary antibody	Dilution		Company	Catalog No.
Goat Anti-Rabbit	1:200		Beyotime	A0208

Supplementary Table 2. Primers used in qRT-PCR.

Gene	Forward primer sequence (5'-3')	Reverse primer sequence (5'-3')
RFWD3	AGTTGGCGTAGGTGCATTCG	AGCCATCACTGAAACCAGCA
HDAC4	CGGACGCCTCTGTTCAACTT	AGCTCCAAACTCCCACCAA
BIRC5	TCTCAAGGACCACCGCATCT	TTTGCATGGGGTCGTCATCT
RAD51	CAGTGATGTCCTGGATAATGTAGC	TGTTCTGTAAAGGGCGGTGG
BRCA1	TGGCAACATACCATCTTCAACC	TGTCAATTCTGGCTTCTCCCT
CCNB1	AAACTTTGGTCTGGGTCGGC	TGCTGCAATTTGAGAAGGAGG
CCNB2	AAGTTCCAGTTCAACCCACCA	GCAGAGCAAGGCATCAGAAAA
SKP2	ATAGAAGTGTCCACCCTCCACG	CACCCAGAAAGGTTAAGTCGC
CCNE2	TTGGCTATGCTGGAGGAAGTAA	TTCAGTGCTCTTCGGTGGTG
CDC25B	GACACGCCCGTGCAGAATAA	TGGTCACTGTCCAGGAGGTT
CDC25C	GAAACTGGTCACCTGGATTCTTC	AACCATTCGGAGTGCTACAAAG
CDK1	CCATACCCATTGACTAACTAT	ACCCCTTCCTCTTCACTTTC
TOPBP1	TGAATCTAACATCGCAAACATC	CACAGCATAATCCGCAACAG
CDK4	CTACCAGATGGCACTTACACCC	GCAAAGATACAGCCAACACTCC
WEE1	ATTTGATGTGCGACAGACTCCT	ACTGGCTTCCATGTCTTCACC
CDKN3	TGTGGAATTATCACCCAT	TTAAGGCAGGTTGTAAGC
CHEK1	TTGGCTTGGCAACAGTATTTCG	CCAGCGAGCATTGCAGTAAGT
CLSPN	CTAATCCAGCAGCCAAAC	TTCCTTTCCATCAGTGCC
E2F1	CACTTTCGGCCCTTTTGCTC	GTGCTCTCACCGTCCTACAC
ELAVL1	AGGGGTGACATCGGGAGA	GCTGCTGAACAGGCTTCG
FANCD2	GGCTTGACAGAGTTGTGGATG	TAGGATCTCAGGTAGGCTGGTG
GAPDH	TGACTTCAACAGCGACACCCA	CACCCTGTTGCTGTAGCCAAA
